# Intestinal Fructose and Glucose Metabolism in Health and Disease

**DOI:** 10.3390/nu12010094

**Published:** 2019-12-29

**Authors:** Beatriz Merino, Cristina M. Fernández-Díaz, Irene Cózar-Castellano, German Perdomo

**Affiliations:** 1 Instituto de Biología y Genética Molecular-IBGM (CSIC-Universidad de Valladolid), Valladolid 47003, Spain; bea.merino.antolin@gmail.com (B.M.); cristinamariafernandezdiaz@gmail.com (C.M.F.-D.); gmperdomo@ubu.es (G.P.); 2 Centro de Investigación Biomédica en Red de Diabetes y Enfermedades Metabólicas Asociadas (CIBERDEM), Madrid 28029, Spain; 3 Departamento de Ciencias de la Salud, Universidad de Burgos, Burgos 09001, Spain

**Keywords:** fructose, glucose, small intestine, liver, brain, gut-brain axis, non-alcoholic fatty liver, insulin resistance, metabolic syndrome, type 2 diabetes mellitus

## Abstract

The worldwide epidemics of obesity and diabetes have been linked to increased sugar consumption in humans. Here, we review fructose and glucose metabolism, as well as potential molecular mechanisms by which excessive sugar consumption is associated to metabolic diseases and insulin resistance in humans. To this end, we focus on understanding molecular and cellular mechanisms of fructose and glucose transport and sensing in the intestine, the intracellular signaling effects of dietary sugar metabolism, and its impact on glucose homeostasis in health and disease. Finally, the peripheral and central effects of dietary sugars on the gut–brain axis will be reviewed.

## 1. Introduction

According to the World Health Organization, obesity is the epidemic of the 21th century. About 13% of the world’s adult population is obese [1]. Worldwide, between 1975–2016, the global obesity rate was nearly triplicated, increasing from 1% up to 6%–8% among children and adolescents [1]. As a major public health issue, clinical interventions based on low-fat diets attracted significant interest. However, over decades, the consumption of sugars has risen significatively worldwide, and has been partially associated to the rapid increase in the prevalence of obesity [2].

From the Industrial Revolution, the consumption of sweeteners has increased dramatically, causing a dietary switch in the world population [3]. Most of this increase in sugar consumption is derived from refined or processed fructose, which is obtained from the conversion of glucose from sugar cane and corn through a chemical process developed in 1957 [4,5]. Fructose constitutes a significant portion of the caloric intake in many countries [3]. The average daily consumption of added sugars (13%–17% of daily energy intake), of which about half is fructose, is above the recommended limit of 10% in many countries [6]. Of note, 16% of total energy in children’s diets comes from added sugars [7]. The increase in total fructose intake parallels a decrease in the proportion of dietary fructose coming from fruits, but augmented proportion from fructose-based sweeteners [3]. In the past, fructose was considered sweeter, more soluble, and less gluconeogenic than glucose and sucrose, and was proposed as a substitute for these sugars [8]. Over time, this idea has been reconsidered in view of the impact of high-fructose consumption on whole-body metabolism, and because it is a risk factor for developing obesity and diabetes [9,10,11].

Although fructose and glucose share the same molecular formula (C_6_H_12_O_6_) and caloric value (4 kcal/g), fructose tastes sweeter than glucose (relative to sucrose, which by consensus agreement is equal to one; the sweetness of glucose is 0.75, and fructose is 1.7), and has a lower glycemic index than glucose (23 versus 100, respectively) [12]. In addition, fructose is less satiating than glucose, increasing food intake [13]. As reviewed in detail below, intestinal fructose and glucose absorption are also quite different, because glucose transport is an energy-requiring process mediated by the sodium-glucose co-transporter 1 (SLGT1), whereas fructose moves through a facilitated passive transport mediated by GLUT5 [14]. Furthermore, fructose metabolism has a negligible impact on circulating insulin levels compared to glucose metabolism, which is related to insufficient leptin (the satiety hormone) secretion, and suppression of ghrelin (the hunger-promoting hormone) [15].

## 2. Intestinal Fructose Transport and Metabolism: Implications for Health and Disease

Whole body fructose homeostasis results from two main processes: Intestinal absorption and clearance, the latter is commonly assumed to be mainly mediated by the liver (~55%–71%) and, to a lesser extent, by kidneys (<20%) [16]. Dietary fructose moves from the intestinal lumen to the circulation through a facilitated passive transport [3] across enterocyte membranes by members of the facilitative glucose transport (GLUT; Slc2a) family [14]. Upon its intestinal absorption, fructose reaches the liver through the hepatic portal vein and undergoes metabolization in hepatocytes [16]. Fructose transport and metabolism has been extensively reviewed (see refs. [3,14,17]). Exhaustive description of fructose hepatic metabolism is out of the scope of this review. Here, we briefly describe the regulation of intestinal fructose transport and transporters and its intracellular metabolism. We focus on revisiting the role of the liver and small intestine in fructose clearance, the relevance of endogenous fructose production in human diseases, and plant extract inhibitors of fructose transporters.

### 2.1. Intestinal Fructose Transport

**Figure 1:**
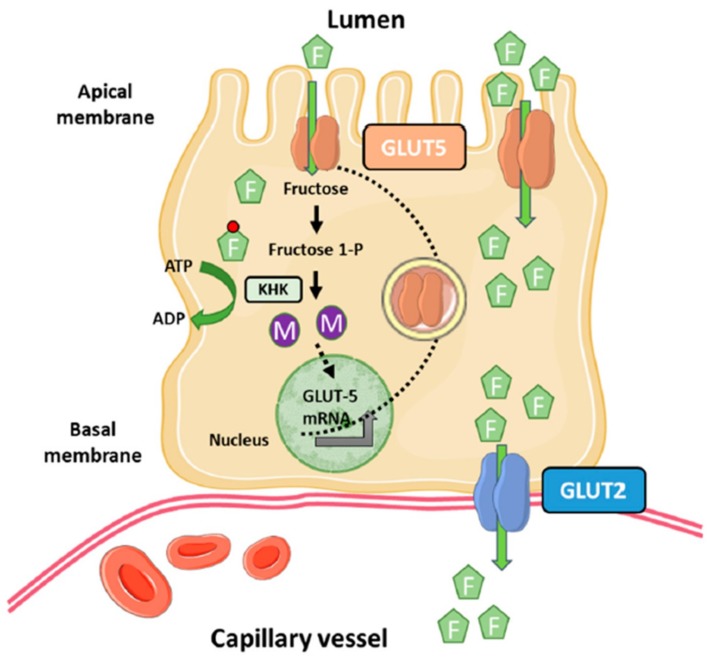
Fructose transport in the small intestine. Fructose (**F**) is transported by GLUT5 through the brush border membrane and enters cytosol of enterocytes, where it is rapidly phosphorylated by ketohexokinase (KHK), leading to a rapid depletion of intracellular ATP levels. A pool of phosphorylated fructose is partially or totally metabolized yielding metabolites (**M**) that induce *Slc2a5* gene expression. The remaining fructose is released across the basolateral membrane into portal circulation down the concentration gradient by GLUT2. In this conceptual model, the small intestine passively transport fructose to portal circulation and the liver is a major organ for fructose metabolism. This figure was created using Servier Medical Art (available at https://smart.servier.com/).

Fructose uptake into enterocytes is an insulin-independent process [18]. Among the members of the GLUT family able to transport fructose (GLUT5, GLUT8 and GLUT11), GLUT5 (*Slc2a5*) is primarily responsible for fructose uptake into the enterocyte at the apical side of the membrane, whereas GLUT2 (*Slc2a2*) moves most of fructose from the cytosol into blood vessels at the basolateral side of the enterocyte [14,19,20,21] (Figure 1). Although GLUT5 belongs to the GLUT family, it only transports fructose without the ability to transport glucose or galactose. Conversely, GLUT2 can transport glucose and galactose in addition to fructose, with an affinity (*K_m_*) for fructose more than five-fold higher than that of GLUT5 [22,23].

The main site of GLUT5 expression is the apical membrane of intestinal epithelial cells, although to a much lower extent is also expressed in kidneys, brain, fat, testes, and muscle [24]. However, the physiological relevance of GLUT5 expression in these extraintestinal human tissues is uncertain. On the other hand, GLUT2, in addition to the basolateral membranes of intestinal epithelial cells, is highly expressed in hepatocytes, pancreatic β-cells, and the basolateral membranes of kidney epithelial cells [22].

The *K_m_* of GLUT5 for fructose varies depending on the study model and the species used for its assessment. Thus, Burant et al. reported a *K_m_* of ~6 mM in *Xenopus* oocytes expressing the mammalian GLUT5 [19]. In contrast, Kane et al. reported a *K_m_* of 11–15 mM using the same expression system for human GLUT5 [25]. Similar values (*K_m_* of 11–13 mM) were found for mouse and rabbit GLUT5 transporter expressed in oocytes [26,27]. Finally, Mate et al. reported a *K_m_* of ~8–11 mM in ileal brush border membrane vesicles of normotensive Wistar-Kyoto rats and their spontaneously hypertensive rats [28]. Assuming a *K_m_* value ranging from 11–15 mM for GLUT5, this *K_m_* is similar to that reported for intestinal luminal fructose concentrations (26 mM) in rats fed dietary fructose [29]. On the other hand, the *K_m_* of GLUT2 for fructose is ~11–17 mM [22,23].

### 2.2. Dietary Fructose Metabolism

High concentrations of dietary fructose in foods and drinks lead to elevated intestinal luminal fructose concentrations that are needed for driving the facilitated fructose transport across the enterocyte membrane, and fluctuate around the *K_m_* of GLUT5 for fructose [3]. Unlike the high fructose concentration in luminal small intestine, fructose concentrations in systemic circulation are relatively low as a result of intestinal absorption and liver clearance rates. In humans, estimates of fasting systemic blood fructose concentrations are low (<0.05 mM), even in those healthy humans consuming high-fructose or sucrose diets (~0.2–0.5 mM) [30,31,32,33], which is still very low compared to fasting blood glucose levels (5.5 mM). Finally, type 1 and type 2 diabetic patients exhibited 0.016 mM and 0.009–0.013 mM fasting fructose concentrations, respectively [34,35]. The low fructose concentrations in peripheral blood support the notion that the liver and kidneys are much more sensitive to small changes in circulating fructose levels than the small intestine. Nonetheless, it is unclear how hepatocytes or nephrons reabsorb fructose from the sinusoidal capillaries or glomerular filtrates, respectively, containing very low fructose levels.

Metabolization of dietary fructose in the small intestine is a process regulated at various steps. In the first step of the classical Hers pathway for fructose metabolism, fructose is mobilized from intestinal lumen into the cytosol of enterocytes by GLUT5, where it is rapidly phosphorylated by the ketohexokinase (KHK, *Khk*), also known as fructokinase, to fructose-1-phosphate using ATP as a phosphate donor [36]. The *Khk* gene encodes two isoforms of the enzyme as a result of alternative splicing of the adjacent exons 3A and 3C of the gene leading to the KHK-A and KHK-C isoforms, respectively [37,38]. Studies of expression analysis in several human and rat tissues indicated that only one mRNA variant is expressed in each tissue [38], but the pancreas is an exception to this pattern because, although KHK-C expression predominates, some KHK-A is also expressed. The KHK-C mRNA variant is expressed at high levels in the liver, kidneys, and duodenum, and is considered more physiological than the KHK-A variant because its *K_m_* for fructose is <1 mM [39,40]. On the other hand, KHK-A is expressed at a low level in a wide range of tissues including skeletal muscle and adipose tissue [39,40]. KHK-A *K_m_* for fructose is 8 mM, suggesting that it poorly phosphorylates fructose at physiological concentrations and that it may have a more important role when fructose intake is excessive [41].

In the second step of the fructose pathway, fructose-1-phosphate is split into glyceraldehyde and dihydroxyacetone phosphate by aldolase B (ALDOB; *Aldob*) [16]. In the third and final step of the pathway, the triokinase (TKFC; ATP:D-glyceraldehyde 3-phosphotransferase) catalyzes the phosphorylation of glyceraldehyde by ATP to form glyceraldehyde-3-phosphate [16,36]. Both ALDOB and TKFC are highly expressed in the liver, kidneys, and small intestine, relative to other organs [42].

Unlike glycolysis, the catabolism of fructose (fructolysis) bypasses major regulatory steps of glycolysis and gluconeogenesis (i.e., phosphofructokinase and fructose-1,6-bisphosphatase), and it is not regulated by feedback inhibition [16,43]. In addition, fructolysis bypasses the glucose-6-phosphate and fructose-6-phosphate production from the pentose phosphate pathway for de novo synthesis of nucleotides and nucleic acids [44]. Thus, it is plausible that in conditions of excessive fructose consumption, KHK-mediated fructolysis leads to increased glyceraldehyde, dihydroxy-acetone-phosphate, and glyceraldehyde-3-phosphate production, which are the source of gluconeogenic and lipogenic substrates (e.g., pyruvate, lactate, acetyl-CoA, and glycerol-3-phosphate), leading to elevated rates of gluconeogenesis, glycogenesis, and/or lipogenesis. Another consequence of the fructolysis is a rapid ATP and Pi intracellular depletion [45,46].

### 2.3. Regulation of GLUT5

In addition to dietary fructose catabolism, metabolism of fructose comprises its biosynthesis from glucose through the polyol pathway [16]. This two-step pathway becomes active when intracellular glucose concentrations are elevated. In the first step, glucose undergoes reduction by NADPH to sorbitol (polyol) by the rate-limiting enzyme in the pathway, the aldose reductase (AR), followed by metabolization of sorbitol into fructose by sorbitol dehydrogenase (SDH) in the presence of NAD+ as a cofactor [16].

Intestinal fructose metabolism is not only important for the metabolic fate of fructose but for the up-regulation of GLUT5, KHK, ALDOB, TKFC, fructose-1,6-bisphosphatease, and glucose-6-phosphate (Figure 1) [47,48]. Thus, it has been extensively shown that chronic or acute fructose exposure increases GLUT5 levels and activity in rodents and human proximal intestine regions [3,17]. The response of GLUT5 to its substrate requires partial or total metabolization of fructose because the nonmetabolizable fructose analog 3-*O*-methylfructose has a modest effect on GLUT5 expression [49], and blocking intracellular fructose metabolism in the HKH^-/-^ mouse model prevents fructose up-regulation of GLUT5 [47]. Furthermore, these effects of fructose on GLUT5 expression are very specific because fructose, glucose, and nonmetabolizable glucose analogs have similar changes on GLUT2 expression in intestinal cells [49]. The molecular mechanisms underlying fructose-mediated regulation of GLUT5 in enterocytes remain incompletely understood. In rats, fructose-induced cAMP stimulates fructose uptake without affecting transcriptional regulation of *Slc2a5* [50], whereas in human Caco-2 cells, fructose increases *Slc2a5* mRNA stability mediated by the cAMP pathway [51]. On the other hand, the use of inhibitors or activators of the phosphatidylinositol 3-kinase (PI3K) and/or protein kinase B (PKB) have demonstrated that this signaling pathway mediates the fructose-induced increase in fructose transport without affecting transcriptional regulation of GLUT5 [52]. How does the PI3K/AKT signaling pathway mediate the effects of fructose on GLUT5 upregulation? It is known that Class II PI3Ks control the endocytic trafficking of transporters through the production of phosphatidylinositol 3-phosphate (PtdIns3*P*). This second messenger is required for Rab11 activation, a small GTPase of the Rab family that coordinates endosome recycling to the plasma membrane [53]. Enterocyte-specific Rab11a^ΔIEC^ ablation (Rab11a-KO mouse model) blunted fructose-induced upregulation of GLUT5 in the small intestine, most likely by impairing endosomal trafficking of the fructose transporter towards the apical membrane of the enterocyte [47].

The expression of GLUT5 in the intestine can also be regulated by the carbohydrate response element-binding protein (ChREBP), a liver glucose-responsive basic helix-loop-helix-leucine zipper transcriptional factor [54]. High fructose diet feeding increases intestinal ChREBP protein levels, accompanied by increased fructose transport (GLUT5), fructolytic (fructokinase, ALDOB, TKFC, and lactate dehydrogenase) and gluconeogenic (glucose-6-phosphatae and fructose-1,6-bisphosphatase) gene expression in mice [55]. Conversely, genetic ablation of ChREBP (ChREBP-KO mice) leads to fructose intolerance due to insufficient induction of these genes involved in fructose transport and metabolism [55,56,57,58,59]. The molecular mechanism by which fructose mediates ChREBP-induction of *Slc2a5* gene expression involves direct interaction of ChREBP with the promoter of *Slc2a5* [55] in mice, whereas ectopic co-expression of ChREBP and its heterodimer partner Max-like protein X (MLX) binds to carbohydrate response elements (ChoREs) and activates *Slc2a5* promoter in Caco-2BBE human cells [55]. Further work is required to confirm whether, similarly to glucose, fructose might regulate ChREBP activity by posttranslational modifications such as *O*-glycosylation, phosphorylation and conformational changes in intestinal cells [57].

Another identified regulatory protein of intestinal fructose transport is the thioredoxin-interacting protein (TXNIP, *Txnip*), an arrestin-like protein that can bind to thioredoxin protein that regulates cellular metabolism and redox state [60,61]. In response to glucose, the transcriptional complex ChREBP/MLX and MondoA/MLX binds to the ChoRE on the *Txnip* promoter to induce mRNA expression [62,63]. Glucose-induced TXNIP inhibits glucose transport through interaction with GLUT1 and inducing its internalization through clathrin-coated pits, as well as reducing the expression of GLUT1, whereas energy stress leads to TXNIP degradation through phosphorylation by AMP-dependent protein kinase (AMPK), resulting in increased GLUT1 function and mRNA expression [61,64]. Dotimas et al. demonstrated that TXNIP regulates fructose absorption in the small intestine [65]. Although the precise mechanisms remains elusive, TXNIP is upregulated in response to fructose consumption and co-immunoprecipitates with GLUT2 and GLUT5. It may be possible that the link between fructose transport and TXNIP may be mediated by phosphorylation of the protein mediated by AMPK, similar to what we described above for GLUT1 [65].

The expression of GLUT5 and its activity is also regulated by early development in the intestine of mammalians (i.e., rat, rabbit, and humans). In rats, under normal conditions (suckling and weaning), intestinal fructose transport and GLUT5 mRNA levels are very low due to the fact that maternal milk is fructose-free, unless there is a precocious exposure to luminal intestine fructose signal, which in turn stimulates GLUT5 expression and activity [17]. The mechanism by which fructose increases GLUT5 expression and activity during weaning is complex and involves systemic levels of glucocorticoids, but not thyroxine [17,66,67,68]. In addition, the diurnal rhythm regulates GLUT5 mRNA and protein expression in adult rats, but this regulation is not present in neonates [69]. Independently of fructose uptake, 3–4 h before the onset of peak feeding, GLUT5 levels increase by four-fold. This diurnal rhythm is also accompanied by upregulation of GLUT2 [8].

### 2.4. Fructose Metabolism in Human Diseases

Major pathways of fructose metabolism are conversion to glucose and lipids [16]. Therefore, excessive fructose intake would result in increased portal fructose concentrations that stimulates endogenous glucose production and lipid synthesis in the liver, which is associated with metabolic syndrome (MetS) [70,71,72], non-alcoholic fatty liver disease (NAFLD) [73,74,75,76,77,78,79,80,81], obesity, and type 2 diabetes mellitus (T2DM) [9,10,11,82,83,84,85,86,87,88]. Although there is mounting epidemiological and experimental evidence linking fructose consumption to metabolic diseases, the relative contribution of fructose to these human diseases remains controversial [87,89,90,91].

### 2.5. Revisiting the Role of Liver and Small Intestine in Fructose Clearance

Traditionally, the liver has been considered as the main organ that metabolizes fructose before entering systemic circulation [16]. This assumption is based on the following evidences: (1) Intestinal absorption of fructose is primarily driven to the liver through portal circulation; (2) peripheral tissues, such as skeletal muscle, have low capacity for fructose metabolism; and (3) the ketohexokinase isoform KHK-C is expressed at the highest level in the liver relative to extrahepatic tissues, leading to a high capacity for fructose phosphorylation and extraction from the blood. In this way, the liver would prevent high fructose doses to spill over peripheral tissues [16].

**Figure 2:**
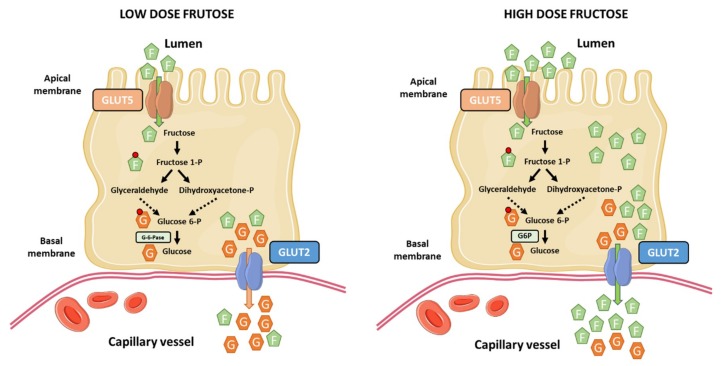
Current view of intestinal fructose metabolism in mice. Under conditions of low-dose dietary fructose (**F**) consumption, most of the fructose is metabolized by the Hers pathway appearing into portal circulation mainly as glucose (**G**), and the remaining as unmetabolized fructose. Conversely, under high-dose dietary fructose consumption, intestinal capacity is overwhelmed, leading to most of the fructose to spill over to the liver. Further work is needed to validate or refute this model in humans. G-6-Pase; glucose-6-phosphatase. This figure was created using Servier Medical Art.

The current notion that the liver is the main site of dietary fructose metabolism and clearance has been recently challenged by Jang et al. [92]. They used sophisticated and elegant isotopic tracing techniques and arterio-venous blood sampling to demonstrate that most ingested fructose is metabolized by the small intestine in mice. At low-doses of fructose (<0.5 g kg^−1^), ~90% of fructose phosphorylation occurs in the jejunum, duodenum, or ileum. Most of this fructose is metabolized in the small intestine, appearing in the portal circulation as glucose and lactate (~60%), and the remaining as fructose (<20%). In contrast, high-doses of fructose (≥1 g kg^−1^) saturate the absorption and catabolism of fructose in the small intestine, leading to fructose spill-over into the liver (>30%) and the colonic microbiota in mice [92] (Figure 2). This work challenges our current knowledge about the role of the small intestine in dietary fructose metabolism and spurs the notion that the small intestine shields the liver from toxic fructose exposure. However, several questions arise from this work and remain to be fully addressed: (1) A limitation of the study is regarding the dose-response to fructose, which may vary between mice and humans. Humans may saturate the capacity for fructose metabolism in the small intestine at relatively lower doses than mice. It is necessary to understand the associated dose-response pattern in humans. (2) The role of the small intestine in fructose metabolism in mice and humans may have diverged across evolution. In fact, humans have a relative shorter gut and smaller intestinal area than rodents [93]. (3) The long standing view is that the liver and kidneys are the only gluconeogenic organs in humans, but not the small intestine because it does not express glucose-6-phosphatase (G-6-Pase) [16]. This critical issue is important to translate experimental evidences from mice to humans. In this line, one study have shown the expression of G-6-Pase in the small intestine of humans [94], and another one showed some evidence of the existence of a conversion of fructose to glucose in human jejunum [95].

### 2.6. Relevance of Endogenous Fructose Production in Human Diseases

In addition to exogenous fructose, fructose can be synthesized from glucose through the polyol pathway [16,96], which has drawn attention on the potential role of endogenous fructose production in metabolic diseases.

The biosynthetic fructose pathway is constituted by two enzymes; the aldose reductase that converts glucose into sorbitol, and the sorbitol dehydrogenase that converts sorbitol into fructose [16]. Under physiological conditions, this pathway is mostly inactive in the majority of body tissues and organs, which has been associated to lower fasting and postprandial circulating fructose levels [97]. However, this pathway can be activated after ingestion of a drink containing glucose (~30 g) and fructose (~30 g) in healthy individuals. Tracer dilution analysis estimated endogenous fructose production ~ 55 mug kg^−1^·min^−1^. This work evidenced, for the first time, the capacity for endogenous fructose production in humans [97]. Further research demonstrated the presence of an active polyol pathway in tissues other than those involved in metabolizing dietary fructose, such as the human brain [98,99,100]. Numerous studies using animal models have linked the polyol pathway to metabolic alterations such as obesity, insulin resistance, diabetes, diabetic nephropathy, chronic kidney disease, acute kidney injury, blood pressure, and MetS [101,102,103,104]. Nonetheless, although the presence of an active polyol pathway has been described in humans, and mounting evidences obtained in animal models of the importance of this pathway in diseases, its significance in human metabolic diseases awaits further confirmation.

### 2.7. Plant Extracts Inhibitors of Fructose Transporters

As described above, multiple studies in humans and animal models have linked fructose consumption with diseases, which has spurred the notion of the potential use of GLUT5 inhibitors for preventing fructose-induced diseases. So far, no potent and specific inhibitors of GLUT5 have been discovered, although phloretin and cytochalasin B are used to inhibit GLUT2 for assessing fructose transport in vitro, whereas GLUT5 is insensitive to both inhibitors [22,25].

In the last decade, plant extracts have been used to screen compounds with inhibitory effects on intestinal GLUT5 transporters. Thus, green tea catechins inhibited D-fructose transport in *Xenopus laevis* oocytes expressing the mammalian GLUT5. Inhibition of D-fructose transport via GLUT5 was more efficient by catechins containing a gallate group [apparent *Ki* values between ~113 and ~117 μM for (−)-epigallocatechin-gallate and (−)-epicatechin-gallate, respectively] than by catechins lacking this group [apparent *Ki* values >500 μM for (−)-epicatechin and (−)-epigallocatechin] [105]. In this line of evidence, it has been shown that chamomile tea and green tea [containing (−)-epigallocatechin gallate (240 mg/g extract), (−)-epigallocatechin (70 mg/g extract), (−)-epicatechin (40 mg/g extract), and (+)-catechin (17 mg/g extract)] effectively inhibited fructose transport through GLUT2 in differentiated Caco-2 cells [106]. In addition, chamomile also inhibits D-fructose transport via GLUT5 in Caco-2 cells and in *Xenopus* oocytes expressing the mammalian GLUT5 [106]. Likewise, Satsu et al. demonstrated that epicatechin gallate inhibited fructose uptake in Caco-2 cells. Interestingly, this reduction in fructose uptake was not related to changes in the affinity (*K_m_*) of GLUT5 for fructose, but with a decrease in the maximal velocity (*V_max_*) [107]. Furthermore, authors demonstrated that epicatechin gallate suppressed fructose permeation in Caco-2 cells, suggesting that this compound suppressed the transepithelial transport of fructose across epithelial cell monolayers, in addition to its effect on fructose uptake. Lastly, authors reported that similar effects on fructose uptake and permeation were observed with nobiletin, another phytochemical tested in this study [107].

An additional compound extracted from the Chinese blackberry tea (rubusoside) inhibited GLUT5-mediated fructose transport in liposomes reconstituted with human GLUT5 purified from insect cells transduced with baculoviruses [18]. Likewise, astragalin-6-glucoside (a glycosylated derivative of astragalin) inhibited GLUT5-mediated fructose transport in these proteoliposomes [18]. The same group performed a virtual screening (in silico) for potential GLUT5 inhibitors using a 3D inward-facing GLUT5 model against a library of >600,000 chemicals [108]. The ability of the top ranked compounds for inhibiting GLUT5-mediated fructose transport were tested in GLUT5 proteoliposomes, identifying the N-[4-(methylsulfonyl)-2-nitrophenyl]-1,3-benzodioxol-5-amine (MSNBA) as an specific inhibitor, which did not affect the fructose transport of human GLUT2 or the glucose transport of human GLUT1-4 [108]. Additionally, whole-cell systems for high-throughput screening of potential GLUT5 inhibitors and activators have been developed using a yeast strain deficient in fructose uptake [109].

The ability of culinary plant extracts containing phytochemicals to inhibit fructose transport has also been assessed in Caco-2 cells. Lee et al. found that demethoxycurcumin and curcumin from turmeric extracts inhibited fructose transport by GLUT2- and GLUT5-mediated fructose uptake, respectively [110]. Similarly, catechin from guava leaf (*Psidium guajava*) inhibited GLUT5-mediated fructose uptake, whereas quercetin inhibited both GLUT5- and GLUT2-mediated fructose transport [110]. In addition, the ability of guava leaf and guava fruit extracts to inhibit glucose transport have also been demonstrated by Müller et al. in Caco-2 cells and mice (C57BL/6N) [111]. The effect of these extracts on glucose uptake in Caco-2 cells were related to inhibition of GLUT2, although the effects on fructose uptake were not assessed [111]. More recently, König et al. demonstrated that fruit extracts prepared from guava inhibited intestinal glucose resorption in a clinical trial [112].

The effects of hesperidin, a flavonoid present in orange juice, on fructose uptake in Caco-2 cell monolayers was studied by Kerimi et al. [113]. They showed that hesperidin inhibited fructose uptake in these cells using fructose (130 mM) as the only source of sugars. Of note, the inhibitory effect of hesperidin on fructose uptake was abolished in the presence of other sugars, such as glucose and sucrose, at high concentrations (120 mM and 130 mM, respectively). Using *Xenopus laevis* oocytes expressing human GLUT2 or GLUT5, they gained insights into the molecular mechanisms by which hesperidin inhibited fructose transport. Thus, hesperidin inhibited the uptake of fructose by GLUT5 expressed in *Xenopus* oocytes. In addition to its effects on fructose uptake, hesperidin lowered glucose uptake in Caco-2 cells and inhibited GLUT2 and GLUT5 transporters when expressed in *Xenopus* oocytes. Lastly, in an attempt to reproduce in vivo these previously observed effects of hesperidin, authors conducted three separated human intervention studies on healthy volunteers using orange juice with different amounts of added hesperidin and a control drink containing equivalent amounts of glucose, fructose, and sucrose, and measured the postprandial glycemic response as biomarker for the effect of hesperidin. They observed that the biggest difference in postprandial blood glucose between orange juice and the control drink was when the juice was diluted [113]. The inhibitory effects of other flavonoids, such as apigenin, on fructose uptake have also been investigated by Gauer et al. in *Xenopus* oocytes. Apigenin, as well as (−)-epigallocatechin gallate, inhibited fructose uptake in oocytes expressing GLUT5 [114].

Finally, acarbose, an α-glucosidase inhibitor that improves insulin sensitivity and decreases postprandial hyperglycemia [115], does not inhibit fructose transport in human Caco-2 cells or in *Xenopus* oocytes expressing the mammalian GLUT2 and GLUT5 [106]. These results suggest that the effects of acarbose on fructose absorption would be mediated by its well-known effects on attenuating sucrose digestion [116], rather than direct effects on fructose transport across the intestinal epithelium.

## 3. Intestinal Glucose Transport and Metabolism: Implications for Health and Disease

Glucose is the main catabolic and anabolic substrate for the great majority of complex organs that controls energy homeostasis in the body. Glucose homeostasis is the result of three physiological events: Intestinal glucose absorption in the post-prandial state, hepatic glucose production (which accounts for ~90% of endogenous glucose production and is the net balance between gluconeogenesis, glycogenolysis, glycogen synthesis, glycolysis, and other pathways), and extrahepatic glucose usage, mainly by the brain, the skeletal muscle, and the adipose tissue. Glucose controls hormonal secretion in endocrine pancreas (i.e., insulin, glucagon, and somatostatin) [117,118,119] and neuronal signaling involved in glucose homeostasis, feeding regulation, and energy expenditure [120].

### 3.1. Intestinal Glucose Transport.

**Figure 3:**
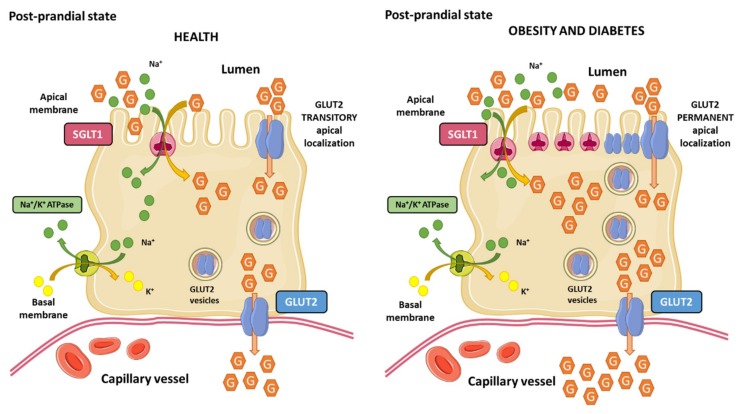
Glucose transport in enterocytes in health and disease. (**Left panel**): After a meal, luminal glucose (**G**) is transported across the apical membrane by SGLT1, and the Na^+^ is then transported out the enterocyte through the basolateral membrane by the Na^+^/K^+^-ATPase. Glucose is phosphorylated and accumulates within the cell. Dephosphorylated glucose passively is transported out of the cell through the basolateral membrane by GLUT2. Alternatively, in response to high glucose luminal concentrations, a pool of endosomal GLUT2 is rapidly and transiently translocated to the apical membrane leading to increased glucose uptake. (**Right panel**): In the setting of obesity and/or diabetes, insulin resistance provokes the loss of GLUT2 trafficking control leading to a permanent localization of GLUT2 in the apical and/or endosomal enterocyte membranes and to increased transepithelial glucose transport from lumen to blood circulation. This figure was created using Servier Medical Art.

Gastric emptying and intestinal glucose absorption determine the glucose appearance rate in the bloodstream after a meal. Intestinal enterocytes are polarized cells responsible for glucose uptake from the intestinal lumen to capillary blood vessels, which is the main mechanism of glucose entrance into the body. Enterocytes express two glucose transporters named sodium-glucose co-transporter 1 (SGLT1; expressed in the brush border membrane) and GLUT2 (localized in the basolateral membrane). SGLT1 couples the transport of one glucose molecule and two sodium ions, which provides the energy to drive glucose accumulation in the enterocyte against its concentration gradient due to the energy stored in the sodium electrochemical potential gradient across the brush border membrane generated by the sodium transport. Sodium is then transported out into the blood vessels by the Na^+^/K^+^-ATPase in the basolateral membrane, maintaining the driving force to transport glucose. As a result, glucose accumulates within the enterocyte and diffuse out of the cell through GLUT2 into the blood stream. This process is ATP-dependent [121,122] (Figure 3).

Intestinal SGLT1 is a high-affinity (*K_m_* ~0.4 mM), low-capacity transporter able to transport glucose or galactose. It is a monomeric integral membrane protein embedded in the lipid bilayer composed by 664 amino acids with 14 transmembrane-spanning regions and it has one glucose binding-site and two sodium binding-sites in the center of the protein. In humans, SGLT1 is encoded by the *Slc5a1* gene, and it is highly expressed in the duodenum and skeletal muscle [123,124]. SGLT1 activity varies diurnally to meet fluctuating availability of glucose. The maximal transport capacity occurs when food is anticipated, and it could be regulated by clock genes [125,126].

After energy-dependent glucose uptake via SGLT1, glucose exits the enterocyte passively through GLUT2 located in basolateral membrane. Intestinal GLUT2 is a facilitative glucose uniporter with low glucose affinity (*K_m_* ~17 mM), but high transport capacity, located in basolateral membrane of the enterocytes. GLUT2 can also transport galactose, mannose, and fructose (with low affinity), and glucosamine with high affinity (*K_m_* ~ 0.8 mM) [127].

### 3.2. Regulation of SGLT1 and GLUT2

SGLT1 expression in the intestinal lumen is regulated by dietary carbohydrate content. Thus, luminal glucose, but not intravenous administration of glucose, increases intestinal SGLT1 expression. High-diet glucose feeding increases SGLT1 expression and activity (rat, mouse, and sheep), which is accompanied by increased glucose transport. Similarly, obese mice exhibit increased intestinal glucose transport mediated by augmented SGLT1 transporters, without increased activity [128,129,130,131,132].

In addition to glucose-mediated regulation of SGLT1, phosphorylation by protein kinase A (PKA) and protein kinase C (PKC) regulates its activity. In humans, SGLT1 contains one consensus site for regulation by PKA and five consensus sites for PKC. The number of consensus sites and conserved sequences varies between species (rat, rabbit, and humans) [133,134]. In Chinese hamster ovary (CHO) cells overexpressing human SGLT1, activation of PKA increased the amount of SGLT1 in the membrane [135]. In contrast, stimulation of human embryonic kidney cells expressing human SGLT1 with 8-Br-cAMP (a brominated derivative of cyclic adenosine monophosphate that activates cAMP-dependent protein kinase) significantly reduced glucose transport [136]. On the other hand, PKC activation in the absence of RS1 increases transport capacity of human SGLT1, while in the presence of RS1, glucose transport is decreased [137].

The adipocyte-derived hormone leptin also regulates SGLT1. Although leptin is not required for intestinal SGLT1 expression, hyperleptinemia or leptin administration drastically reduce intestinal SGLT1 expression. The intracellular signaling pathways by which leptin regulates intestinal SGLT1 remain incompletely understood, but may include PKA, PKC, and the leptin receptor isoform b [138,139]. Finally, as in the case of GLUT5, green tea catechins markedly inhibit SGLT1-mediated glucose transport in the small intestine, being more pronounced by catechins containing a gallate group [(−)-epigallocatechin-gallate and (−)-epicatechin-gallate] than by catechins lacking this group [140].

The classical view of intestinal glucose absorption is underlined by the evidence that SGLT1 is in the apical membrane of enterocytes, while GLUT2 is located exclusively in the basolateral membrane, leading to the transepithelial glucose transport from the lumen into the portal circulation. This classical theory explains glucose absorption at low luminal glucose concentrations (≤10 mM) but it fails to explain the marked increase at glucose concentrations that surpass SGLT1 (≥25 mM) transport capacity. GLUT2 levels are also regulated by glucose concentrations in enterocytes. As part of an adaptive physiological mechanism in response to increased luminal glucose concentrations, caloric demand, and glucagon-like peptide 2 (GLP-2); GLUT2 is rapidly and transiently recruited to the brush border membrane of the enterocyte, leading to a three-fold enhancement of glucose transport [141,142] (Figure 3). This adaptive mechanism that is known as the “GLUT2 translocation” theory, which in addition to other theories, such as the “solvent drag” theory, have been proposed to explain the marked increase in glucose absorption in response to high luminal glucose concentrations [143].

Conversely, it has also been demonstrated that in addition to high luminal glucose concentrations, insulin decreases GLUT2 membrane levels as a result of the internalization of GLUT2 from plasma membranes back into intracellular pools, leading to the inhibition of glucose transport [144]. The regulation of intestinal glucose absorption by insulin is probably another physiological mechanism at the enterocyte level by which the hormone limits sugar excursions in the blood circulation during a sugar-rich meal. This evidence raised the idea that insulin resistance may provoke a loss of insulin-mediated control of GLUT2 membrane trafficking, leading to unleash intestinal glucose absorption upon high-sugar diets consumption. Tobin et al. demonstrated that insulin resistance in mice provoked a loss of GLUT2 trafficking control, where GLUT2 levels remain permanently elevated in the brush border membrane and low in the basolateral membrane of the enterocyte [144]. Ait-Omar et al. investigated the relevance of these previously described mechanisms in the small intestine of morbidly obese insulin resistant humans and lean control subjects. They found that GLUT2 was accumulated in apical and/or endosomal membranes of enterocytes in obese subjects. Interpretation of these findings is complex, but authors proposed that permanent apical GLUT2 localization in obese subjects would mediate blood-to-lumen glucose flux during fasting hyperglycemia, leading to glucose secretion into the intestinal lumen. In contrast, after consumption of a sugar-rich meal, permanent apical GLUT2 localization would provide a large glucose uptake from the intestinal lumen to the portal circulation [145].

### 3.3. Intestinal Glucose Metabolism in Human Diseases

#### 3.3.1. Relevance of Glycemic Index and Glycemic Load for T2DM

The glycemic response (GR) is the appearance of glucose in blood after a meal. It depends on the amount of glucose absorbed, the rate of glucose entry into circulation, the rate of disappearance due to tissue uptake from circulation, and the regulation of hepatic glucose production [146]. Blood glucose concentrations will rise and fall rapidly or slowly depending on the carbohydrate content of food. The glycemic index (GI) is a tool developed to compare the postprandial responses to constants amounts of different carbohydrate-containing food. It is a useful tool for people with diabetes, providing information on the GR that might be expected when a person consumes the quantity of a food containing a fixed amount of carbohydrates [147]. The glycemic load (GL) concept was introduced as a mean of predicting the GR, considering the GI and the amount of available carbohydrate in a portion of the food eaten [148]. Thus, foods have been classified by GI into low (GI ≤ 55), medium (GI 56–69), and high (GI ≥ 70) categories, and classified by GL as being low (GL ≤ 10), medium (GL 11–19), and high (GL ≥ 20). Since these concepts were introduced, numerous studies have been performed to ascertain how GI and GL relate to health and disease. Of note, the American Diabetes Association (ADA) indicated that current knowledge is insufficient to relate low–GL diet with a reduction on diabetes risk, and that it has not been demonstrated that one method of assessing the relationship between carbohydrate intake and blood glucose response is better than other methods [149].

To shed light into this issue, Livesey et al. performed a review meta-analysis of prospective cohort studies for a comprehensive examination of evidence on the dose-response that links GL to T2DM. The analysis concluded that a GL over a dose range of 100 g/2000 kcal, increases the risk of T2DM by 45%, supporting the notion that GL is an important and underestimated dietary characteristic that contributes to the incidence of T2DM [150]. Greenwood et al., in a systematic review and dose-response meta-analysis of prospective studies, showed that there is a protective effect of low dietary GI and GL and risk of T2DM [151]. In addition, two previous systematic reviews concluded that there is evidence of a positive association between both dietary GI and GL and risk of T2DM [152,153]. In summary, despite the fact that epidemiological studies of GI and GL in relation to diabetes risk have yielded inconsistent results, there is important research in support of significantly positive associations between dietary GI and GL and the risk of T2DM, thus reducing the intake of high-GI foods may bring benefits in diabetes prevention.

#### 3.3.2. Regulation of SGLT1 in Diabetes Mellitus

Several studies in rodent models of T2DM and type 1 diabetes mellitus (T1DM) have shown a link between intestinal SGLT1 expression and diabetes. Streptozotozin (STZ)-induced diabetes in mice and rats (STZ; a toxic drug that produces a destruction of pancreatic β-cells causing insulin deficiency and hyperglycemia [154]) produces increased SGLT1 intestinal expression [155]. Likewise, a rat model of T2DM (Otsuka Log-Evans Tokushima Fatty rats) exhibited increased intestinal mRNA expression of SGLT1 associated with impaired glucose tolerance and occurred before the onset of insulin resistance and hyperinsulinemia [156]. Similar results were confirmed in patients with noninsulin-dependent diabetes mellitus where mRNA and protein levels were increased three- to four-fold in brush border membranes of enterocytes in the small intestine [157]. Finally, in morbid obese non-diabetic patients, increased SGLT1 expression in the intestine was found and it correlated with accelerated intestinal absorption [158].

Taken together, these findings are consistent with the concept that SGLT1-mediated glucose absorption in the intestine underlies the rapid post-prandial rise in blood glucose levels observed in obesity and T2DM. This knowledge has prompted the concept that pharmacological inhibition of SGLT1 in the small intestine can lower hyperglycemia by inhibiting glucose absorption and increasing GLP-1. The pharmacological tools that have been used to determine the potential of SGLT1 inhibition include phlorizin (or phloridzin), canagliflozin, LX4211 (or sotagliflozin), LP-925219, KGA-2727, and GSK-1614235 [159]. The use of these inhibitors in rodent models of T2DM and in humans has lent support to this pharmacological approach in the treatment of T2DM, but more studies are needed on long-term safety of SGLT1 inhibition.

## 4. Peripheral and Central Effects of Dietary Sugars in the Gut–Brain Axis in Health and Disease

### 4.1. The Gut–Brain Axis

In the early 20th century, Ivan Pavlov discovered the existence of a close interaction between the gut and the brain. Pavlov observed in dogs how a stimulus associated with feeding induced vagal-dependent gastric acid secretion [160]. Since then, this interaction has been widely described and is enclosed in the term of “gut–brain axis”, a complex bidirectional communication system that maintains constant crosstalk between the gastrointestinal system and the enteric and central nervous system. This intimate connection involves numerous endocrine, immune, and neuronal pathways [161]. Through this complex system, the gut can send modulating signals to the brain via visceral messages that influence emotional and cognitive brain centers producing different psychobehavioural responses [161]. In the other direction, the brain is able to send orders for proper maintenance of gastrointestinal homeostasis (such as by modulating intestinal motility and mucin production) and can also modulate the immune system (such as by modulating cytokine production by mucosal cells) [161].

The gut–brain axis uses mostly four major information carriers to communicate with each other: Neural messages via vagal and spinal afferent neurons, immune mediators carried by cytokines, endocrine signals carried by gut hormones, and microbiota-related factors that reach the brain directly from the blood stream [162,163]. The integration of all these signals allows the maintenance of a large number of vital functions such as the control of food intake and satiety, the repulsion of harmful foods, and the adaptation of our gastrointestinal system to the environment, giving rise in pathological conditions to the sensation of nausea, pain, or even may result in gastrointestinal dysfunction [164,165].

### 4.2. Regulation of the Gut–Brain Axis by Enteroendocrine Cells and Sensing of Intestinal Sugars

Enteroendocrine cells (EECs) form the largest endocrine organ in the body and play a key role in regulating nutrients intake and postprandial metabolism. Following a meal, EECs in the small intestine sense luminal and circulating levels of nutrients, and simultaneously are stimulated by prevailing nutrients through multiple nutrient transporters and G protein-coupled receptors (GPCRs), leading to activation of intracellular signaling pathways that produce secretion of peptides and hormones. These hormones enter circulation and act on multiple distant tissues such as the brain, gallbladder, and pancreas, as well as, on neighboring enteric neurons, endothelial cells, and the gastrointestinal epithelium. Thus, the physiological role of the enteroendocrine system in response to ingested glucose and fructose is to detect nutrients in the intestinal lumen, to monitor energy status of the body, and to elaborate an appropriate response, through the production of more than 30 different hormones and neurotransmitters to control postprandial whole-body metabolic homeostasis [166].

EECs are endoderm-derived epithelial cells widely distributed in the villi and crypts, where they are interspersed between non endocrine cells [166]. The intestinal epithelium is in a constant turnover that is replenished from pluripotent stem cells at the base of intestinal crypts and their progenies migrating up the crypt–villus axis [167]. The spatial distribution and differentiation of EECs is regulated by an interplay of the surface protein Notch and three basic helix-loop-helix transcriptional factors (Math1, Neurogenin 3, and NeuroD), among other factors [167,168]. EECs are classified depending on their morphology and position in the gastrointestinal mucosa into “open-type” with a bottle neck shape and an apical prolongation with microvilli facing towards the intestinal lumen or “closed type” that are located close to the basal membrane, do not reach the lumen of the gut, and lack microvilli [169,170,171]. The open-type cells are activated by luminal content through the microvilli, whereas the close type cells are activated by luminal content indirectly through neuronal or humoral pathways. In both cases, hormones and peptides accumulate into cytoplasmatic secretory granules that are released by exocytosis at the basolateral membrane upon chemical, mechanical, or neural stimulation [170,172].

#### 4.2.1. Fructose-Induced Hormonal Secretion in Intestinal Cells

Using specialized organoid cultures enriched in a single intestinal cell type, primarily enterocytes, Paneth or goblet, but not intestinal stem cells, Kishida et al. demonstrated that fructose can be sensed by absorptive enterocytes and secretory goblet and Paneth cells, but not stem cells [173]. In response to fructose there was an increased expression of fructolytic genes without affecting non-fructolytic gene expression. Sensing was independent of Notch, Wnt, and glucose concentrations in the culture medium, but required fructose uptake and metabolism. Stronger responses were found in more mature enterocyte- and goblet-enriched organoids. Of note, the response to fructose in enterocyte organoids was retained upon forced dedifferentiation to reacquire stem cells characteristics [173].

Fructose increases secretion of human peptide tyrosine tyrosine (PYY), cholecystokinin (CCK), neurotensin, and serotonin (5-HT) in EECs subtypes L, I, N, and enterochromaffin cells (EC), respectively [174,175]. Likewise, fructose stimulates secretion of glucagon-like peptide 1 (GLP-1) from L-subtype EECs in humans, rats and mice, but not glucose-dependent insulinotropic polypeptide (GIP; glucose-dependent insulinotropic polypeptide or gastric inhibitory peptide) [174,176]. On the other hand, fructose induced the secretion of GIP from K-subtype EECs in mice [176] but is unaffected or reduced in rats and humans [174,177].

#### 4.2.2. Glucose-Induced Hormonal Secretion in Intestinal Cells

Oral glucose, but not intravenous glucose, leads to a greater stimulation of insulin secretion and modulation of glucagon secretion in the pancreas. This physiological response to glucose is called the incretin effect, which is due to the release of incretin hormones (GIP and GLP-1) from specialized EECs [178,179]. Of the three signals originating in the gut (glucose, incretins, and neutral signals transmitted by the autonomic nervous system) that regulate pancreatic insulin secretion, the incretin effect makes a substantial contribution to maintenance of glucose homeostasis [178,179].

GIP is secreted in response to glucose by K-cells located in the duodenum and upper jejunum. GIP is synthesized as a precursor pro-peptide (pro-GIP), which is cleaved to GIP by posttranslational processing. GLP-1 and GLP-2 are secreted in response to glucose by L-cells in the small and large intestine, with a gradient from low density in the duodenum to high density in the ileum, but also in the colon and rectum [180,181]. The proglucagon gene is cleaved to GLP-1 and GLP-2 by posttranslational processing. The biological active forms of GLP-1 are GLP-1 [7-36 amide] (amidated GLP-1), and GLP-1 [7,8,9,10,11,12,13,14,15,16,17,18,19,20,21,22,23,24,25,26,27,28,29,30,31,32,33,34,35,36,37] (glycine-extended GLP-1), which are “truncated” forms in comparison to the originally proposed sequences GLP-1 [1-36 amide] by the N-terminal six amino acids [182,183,184]. In pancreatic α-cells, the same proglucagon gene is processed in a different manner, yielding glucagon and a “major proglucagon fragment that is not further processed to GLP-1 and GLP-2 [185]. Finally, in addition to its role in the regulation of pancreatic insulin secretion, GLP-1 and GLP-2 promotes nutrient absorption [180].

5-HT is secreted in response to glucose by EC cells located throughout the gastrointestinal tract, and regulates intestinal motility and brain control of appetite [186,187]. 5-HT and GLP-1 activate 5-HT_3_ and GLP-1 receptors in the intestinal vagus nerve, respectively, leading to vagal reflexes, which in turn slow the subsequent emptying of carbohydrates from the stomach and induce satiation [188,189].

#### 4.2.3. Intestinal Sweet Sensing and Glycemic Control

The gastrointestinal tract is a major determinant of metabolic homeostasis. Sensing of nutrients, and particularly glucose, in the EECs provides feedback signals from the intestine to slow the rate of gastric emptying, limit postprandial glycemic excursions, and induce satiation.

Intestinal sweet sensing is regulated by the sweet taste receptor (STR), which has been described on K-cells, L-cells, and EECs in humans. Additionally, STRs have been described in metabolic tissues that sense and respond to carbohydrates, such as hypothalamic neurons, hepatocytes, adipocytes, and β-cells (for review see refs. [190,191,192,193]). STR senses hexose sugars, D-amino acids, sweet proteins, and low-calorie sweeteners. The receptor is comprised of a heterodimer of class C, G-protein coupled receptors T1R2 and T1R3. The mechanisms of sweet taste transduction have been mostly studied in lingual sweet taste cells (this topic is out of the scope of this manuscript, for review see refs. [194,195,196,197]). Briefly, the interaction of sweet tastings with STR initiates the dissociation of the gustducin (the G-protein) into Gα and Gβγ subunits and activation of phospholipase C. Then, intracellular Ca^2+^ is released from inositol 1,4,5-triphosphate (IP3)-sensitive stores, leading to opening of the melastatine type-5 transient receptor potential cation (TRPM5) channel allowing sodium influx. Increases in intracellular Na^+^ and Ca^2+^ levels lead to depolarization of the basolateral membrane, which via 5-HT and ATP-dependent pathways activate intermediary taste cells and nerves involved in lingual sweet taste that convey information centrally to the cortex.

Numerous studies in rodents and human cells support the notion that intestinal STR is a glucose sensor on the gut luminal membrane responsible for the regulation of SGLT1 expression and GLP-1 secretion. First, it was demonstrated that T1R2, T1R3, and the α-subunit of gustducin were co-expressed in K- and L-endocrine cells in rodents and humans [198], and to a lesser extend in EC cells containing serotonin in pig intestine [199]. Second, Parker et al. proposed that secretion of GLP-1 by L-cells and GIP by K-cells was through uptake of glucose by SGLT1, suggesting that SGLT1 was likely the mediator of the direct responsiveness of K- and L-cells to luminal glucose [200]. Third, genetic deletion of T1R3 or gustducin in mice abolished the ability of mouse intestine to upregulate SGLT1 expression in response to increased dietary carbohydrate, providing convincing evidence for the involvement of the STR in intestinal sweet transduction [198]. Fourth, genetic deletion of T1R3 and gustducin exhibited deficiencies in secretion of GLP-1 [201]. Fifth, luminal glucose above a threshold results in secretion of GPL-1, GLP-2, and GIP through a signaling pathway involving STR in enteroendocrine cells [198]. These evidences beg an important question: How does glucose activation of STR in EECs cause increased expression of SGLT1 in enterocytes? The communication between EECs and neighboring enterocytes likely resides in the involvement of intermediaries such as GLP-1 and/or GLP-2 and enteric neurons. Thus, GLP-2 receptors are present on enteric neurons [202,203], while enterocytes respond to GLP-2 in an enteric neuron-dependent manner [203]. In addition, GLP-2 upregulates SGLT1 expression [204,205,206], and STR-dependent release of GLP-1 and GLP-2 is detected at higher concentrations in the portal and lymphatic circulation in rodents [204,207].

**Figure 4:**
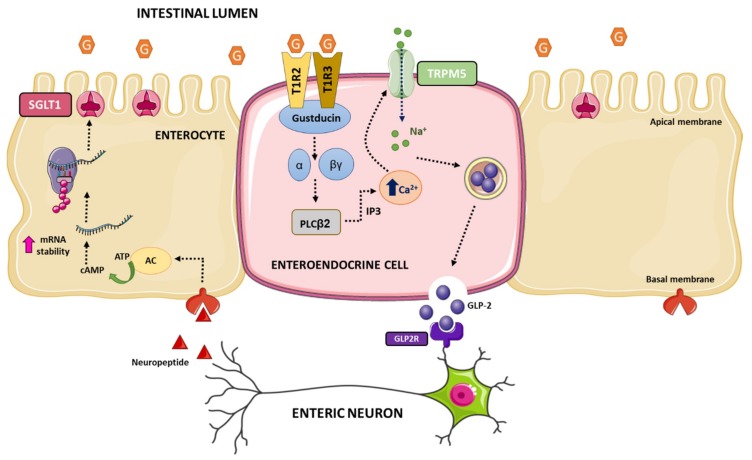
Model of intestinal glucose sensing and signaling pathways. Glucose (G) binds and activates the taste receptor type 1 member (STR) comprised of a heterodimer T1R2+T1R3 and G-protein gustducin, leading to its dissociation into Gα and Gβγ subunits and activation of phospholipase C β_2_ (PLCβ_2_) in enteroendocrine cells. Inositol 1,4,5-triphosphate (IP3) triggers intracellular calcium release resulting in increased sodium flux through type-5 transient receptor potential cation channel (TRPM5). Depolarization of the basal membrane results in GLP-2 release, which triggers release of an unidentified peptide from enteric neurons at neighbor enterocytes. Signal transduction in enterocyte leads to cAMP-mediated adenylate cyclase (AC) production that increases stabilization of *Slc5a1* mRNA and ultimately augmented translation and insertion of SGLT1 into apical membrane. This figure was created using Servier Medical Art.

All these evidences have led to a model of intestinal dietary glucose sensing. Luminal glucose is sensed by STR expressed on the luminal membrane of enteroendocrine cells. Above a threshold level of luminal glucose, the hexose binds to and activates STR, initiated by dissociation of gustducin into Gα and Gβγ subunits, which leads to activation of phospholipase C β_2_. Then, IP3-sensitive stores release intracellular Ca^2+^ that opens the TRPM5 channel increasing sodium influx. Intracellular elevation of Ca^2+^ and Na^+^ depolarizes the basolateral membrane resulting in the release of GLP-2. GLP-2 binds to its receptor on enteric neurons evoking an action potential that triggers the release of an unknown neuropeptide to the vicinity of neighboring enterocytes. The neuropeptide binds to its receptor located on basolateral membranes of enterocytes leading to a rise in intracellular levels of cAMP, which increases stabilization of the 3′end of *Slc5a1* mRNA and ultimately augmented SGLT1 translation and insertion into the apical brush border membrane of the enterocyte (Figure 4).

### 4.3. Central Effects of Glucose and Fructose Consumption

Sugar overconsumption has been associated with detrimental metabolic effects, such as obesity, dyslipidemia, MetS, and impaired insulin sensitivity [71,208,209]. Therefore, it is necessary to understand the specific molecular mechanisms by which dietary sugars cause an addictive eating behavior and how sugar intake affects the gut–brain axis. Herein, we will review the effects of the two main dietary monosaccharides: Glucose and fructose, the latter of which is usually consumed in the form of sucrose disaccharide (50% glucose, 50% fructose) or in the form of high-fructose corn syrup (HFCS) (range 47%–65% fructose, and 53%–35% glucose) [210], the major component of sweetened soft drinks.

Appetite control is a complex crosstalk between the periphery and the central nervous system that involves a large number of peptides and hormones [211]. Disturbances in food intake control will ultimately be responsible for large changes in energy balance and different metabolic effects. The appetite regulatory hormones are secreted from peripheral tissues such as the pancreas (e.g., insulin), adipose tissue (e.g., leptin), or the gastrointestinal tract [e.g., ghrelin, CCK, PYY, GLP-1 and GIP], and bind to receptors located in the arcuate nucleus of the hypothalamus, where they inhibit or stimulate appetite or satiety [212].

Many studies have demonstrated that circulating levels of satiety hormones are regulated by the type of sugar consumed. In response to glucose stimuli, a cascade of hormonal secretion is triggered. Thus, glucose produces a repression of the hunger hormone ghrelin (secreted by the stomach), whereas there is a stimulation of the secretion of satiety hormones such as leptin, insulin, GIP, GLP-1, and PYY. However, fructose produces lower repression of ghrelin and a decreased stimulation of satiety hormones (leptin, insulin, GIP, GLP-1, and PYY) than glucose [13,83,212,213,214,215,216,217]. These effects may be related to different explanations such as the lower ratio of intestinal fructose uptake, the lower intestinal levels of GLUT5 compared to the high levels of GLUT2, and also due to the low expression of GLUT5 in pancreatic β-cells leading to decreased insulin release [218,219,220].

**Figure 5:**
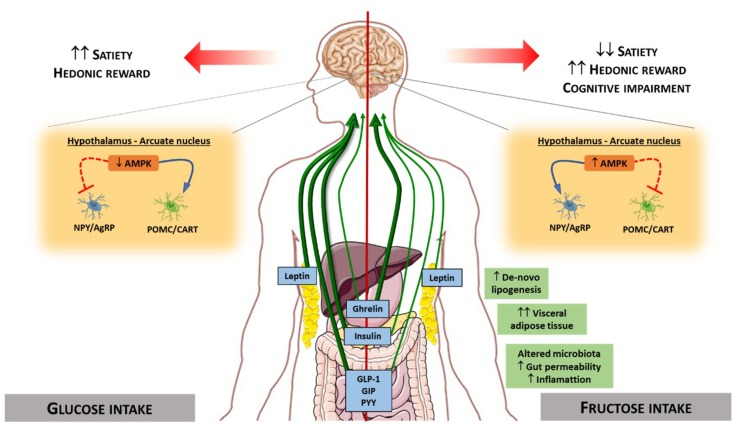
Peripheral and central effects of glucose and fructose on food intake. Upon nutrient ingestion, glucose triggers the secretion of anorexigenic peptides such as leptin, insulin, GLP-1, GIP, PYY, and blocks the secretion of the orexigenic hormone ghrelin. These peptides will induce AMPK inhibition, which will lead to the stimulation of POMC/CART neurons, contributing to satiety response. However, upon fructose consumption, the secretion of anorexigenic peptides is decreased, as well as the repression of ghrelin secretion. These facts will cause the increase in AMPK activity, which will lead to the repression of POMC/CART neurons and the activation of NPY/AgRP, leading to reduced satiety suppression, as well as higher hedonic reward to food and increasing food intake, in addition to playing a possible role in cognitive functions. This figure was created using Servier Medical Art.

Some of these hormones regulated differentially by fructose or glucose convey signals to brain structures. Specifically, there are two neuronal types in the arcuate nucleus that integrate signals from the periphery, acting as metabolic sensors: Neurons co-expressing agouti-related peptide (AgRP) and neuropeptide Y (NPY), whose activation triggers orexigenic effects; and neurons expressing pro-opiomelanocortin (POMC), whose activation triggers anorexigenic effects [221,222,223]. These different types of neurons are sensitive to changes in hormone levels promoting or suppressing food intake. Therefore, the differential effect of dietary sugars on hormonal levels affects neuronal stimulation causing both short-term and long-term central effects in the regulation of food intake and energy homeostasis [224,225]. The low stimulatory capacity of fructose on satiety hormones such as leptin and insulin will lead to low stimulation of POMC neurons and the maintenance of the signal on NPY/AgRP neurons, thus promoting less satiety than glucose, and therefore increased food intake. In the same way, the hypothalamic AMPK functions as a ‘fuel gauge’ to monitor cellular energy status, and its inhibition promotes anorexigenic effect [226]. AMPK activity is inhibited by leptin and insulin. Intracerebroventricular glucose administration in rodents inhibits hypothalamic AMPK activity and suppresses food intake, whereas fructose activates it, thus promoting an orexigenic effect [227,228,229,230] (Figure 5).

With the use of new technological advances, it is possible to evaluate the brain activity produced by the intake of different nutrients. In humans, differences in cerebral blood flow have been reported between subjects undergoing glucose and fructose infusions [231], and compared to glucose, fructose causes poor satiety stimulation in specific appetite-regulating regions (e.g., hypothalamus) [13]. It has also been observed that fructose ingestion compared to glucose resulted in a significantly greater incentive value of food cues [232]. These findings suggest that fructose promotes effects on brain activity that affect appetite, probably promoting less satiety than other sugars in humans.

In addition to the above-mentioned findings, it has been described that high-fructose intake may affect central appetite regulation by altering specific components of the endocannabinoid system in rats. Fructose consumption has been reported to significantly increase the mRNA expression of the cannabinoid 1 receptor (CB1) [233], and induces an increase in fatty acid amide hydrolase (FAAH) and diacylglycerol lipase (DAG) 1β, but a decrease in DAG1α mRNA [234]. These changes in the endocannabinoid system suggest that fructose consumption may lead to increased hedonic reward for food, thus leading to disturbances in the eating behavior pattern.

The consumption of dietary sugars has not only been related to central effects that control appetite and satiety, but also to disturbances in cognitive functions. In rodents, studies have shown that fructose consumption reduced phosphorylation levels of the insulin receptor, leading to impaired brain insulin signaling [235,236], a harmful feature associated with cognitive impairment [237]. Moreover, diminished phosphorylation of cAMP-response element binding and synapsin I, and reduced synaptophysin levels have been observed after fructose intake [236]. Together, these findings indicate that excessive fructose consumption could lead not only to detrimental effects in eating behavior, but also can trigger impaired cognitive function. Further work is required to investigate these evidences.

#### The Fructose Hypothesis

In view of the association between fructose consumption in Western diets and MetS, fructose has been suggested as one of etiological factor of MetS. The “fructose hypothesis” proposed that a high amount of fructose consumption is a leading risk factor for the development and progression of MetS, covering obesity, insulin resistance, dyslipidemia, fatty liver, and cardiovascular disease [238,239,240].

Fructose may cause insulin resistance by accumulation of triglycerides in the liver. There are two metabolic pathways to increased hepatic lipid content, i.e., lipogenesis and/or reduced mitochondrial fatty acid oxidation. Hepatic fructolysis leads to increased gluconeogenic sources resulting in elevated rates of lipogenesis [16,45,46]. Hepatic accumulation of toxic intermediary lipid metabolites, such as diacylglycerol (DAG) results in PKCε activation that impairs hepatic insulin signaling through phosphorylation of serine residues on the insulin receptor substrate 1 and 2 (IRS1/2). When hepatic insulin signaling is impaired, gluconeogenesis and glycogenolysis are unleashed, contributing to hyperglycemia and hyperinsulinemia. Under these circumstances, hepatic lipid synthesis is enhanced due to hyperinsulinemia [241,242]. Likewise, reduced fatty acid oxidation leads to hepatic triglycerides accumulation. Of note, Ohashi et al. demonstrated that excessive amounts of fructose consumption lead to epigenetic modifications, such as DNA hypermethylation of promoter regions of peroxisome proliferator-activated receptor alpha (PPARα) and carnitine palmitoyl transferase 1A (CPT1A) that results in lower amounts of mRNA levels [243]. Hepatic triglyceride accumulation results in augmented secretion of very low-density lipoprotein (VLDL) leading to increased lipid uptake in skeletal muscle and peripheral tissues. Similarly to what happens in the liver, intramyocellular lipid accumulation (particularly DAG) activates the PKCθ isoform that phosphorylates and inactivates IRS1 resulting in impaired insulin-stimulated glucose uptake, contributing to hyperglycemia, increased delivery of glucose to the liver, and hyperinsulinemia [241,242].

Fructose-induced hyperuricemia has also been proposed as a causal agent in the etiology of insulin resistance [244,245,246]. This notion arises from the observation that lowering uric acid levels prevents the development of MetS induced by fructose [244,245,246], defective endothelial NO production in mice leads to development of MetS [247], and that uric acid inhibits endothelial NO in in vitro and in vivo [248]. Two mechanisms have been proposed: The first mechanism proposed is that uric acid inhibits endothelial nitric oxide (NO) release, and NO increases blood flow ensuing enhanced insulin delivery and glucose disposal in skeletal muscle and peripheral tissues [249]. The second mechanism states that uric acid promotes inflammation and oxidative stress within the adipocyte [250,251,252]. In addition, uric acid-mediated insulin resistance in the adipose tissue, via the classical mechanisms (i.e., low-grade chronic inflammation mediated by proinflammatory cytokines secreted by the adipocytes, increased lipolysis, and reduced lipogenesis), may result in MetS [241,242].

Additionally, persistent high fructose consumption leads to higher levels of leptin and leptin resistance, which in turn increases food and energy intake [253]. Potential molecular mechanisms underlying leptin resistance may be related to impaired leptin transport across the blood-brain barrier and/or reduced basal levels of phosphorylated signal transducer and activator of transcription 3 (STAT3; a downstream component of the leptin receptor signaling cascade), despite equivalent expression of leptin receptors, in the hypothalamus [253].

However, the fructose hypothesis is not universally accepted. It has been argued that fructose is rarely consumed in its pure form and many published studies have used fructose levels that far exceed dietary composition [254]. Likewise, many animal studies have used extremely high-fructose doses or unusual glucose to fructose ratio that are not representative of actual human diets, which makes it difficult to extrapolate this phenomenon to humans. Therefore, caution in interpreting studies of the effects of fructose on health should be taken into consideration [254]. Another proposed argument to refute the fructose hypothesis is that the causative role of fructose in increasing the risk for the development and progression of MetS is not fully demonstrated. Carefully designed studies should be performed to tease apart the contribution of each risk factor associated to MetS (e.g., obesity, diabetes, or insulin resistance) from fructose, per se [254].

### 4.4. Peripheral Effects of Glucose and Fructose Consumption

#### Gut Microbiota, Lipid Metabolism, and Liver Disease

The gut microbiota is a complex and dynamic population of microorganisms that, in addition to acting as an immune barrier and protecting against pathogens, plays a crucial role as a metabolic organ itself modulating intestinal permeability, and therefore the nutrient availability [255]. It is generally known that diet exerts a large effect on the gut microbiota, which may affect intestinal permeability and ultimately cause a great metabolic impact [255,256,257,258].

High-fructose or high-glucose diets have been described as an intestinal microbiota modulator that increases inflammation, gut permeability, and metabolic endotoxemia, causing metabolic disturbances such as hepatic lipid accumulation, liver damage, and insulin resistance [258,259]. Likewise, sugar overconsumption also affects lipid metabolism. In obese and overweight subjects, the consumption of glucose-sweetened beverages leads to a lower increase in plasma triglycerides, de novo lipogenesis, and visceral adipose tissue compared to those that consumed fructose-sweetened beverage [71]. However, in rodents, both high-glucose and -fructose diets stimulated similar hepatic lipogenic gene expression [260].

Liver is the principal metabolic organ within the human body and has a major role in regulating carbohydrate metabolism [261]. Many studies point out to the direct implication of high-sugar diets in the development of serious liver diseases, such as NAFLD, hepatic steatosis, liver fibrosis, and dysfunction [262,263,264]. Multiple studies showed that fructose more potently stimulates hepatic de novo lipogenesis than glucose [78,265,266], and the effect is much higher when both monosaccharides were consumed simultaneously [265]. These differences in de novo lipogenesis between both sugars can be explained by differences in their hepatic metabolism. Fructose is directly phosphorylated by fructokinase, bypassing the enzyme phosphofructokinase, a major rate-limiting step in glucose metabolism, providing a larger available substrate for de novo lipogenesis than glucose [261,267].

Regarding the effect of isocaloric diets with different sugar composition, various studies have observed no differences in liver fat content between high-fructose or high-glucose diets [268,269], nor between isocaloric diets with high-fructose corn syrup or sucrose [270]. However, when comparing different doses of fructose in the diet, liver fat content was increased in high-fructose diet, probably associated with increased de novo lipogenesis and reduced whole-body fatty acid oxidation [266,270]. In the same direction as the previous findings, when comparing hypercaloric diets enriched in either fructose or glucose, no significant changes are observed between both diets, suggesting that high-glucose and high-fructose diets provide the same risk for the development of NAFLD [269,271,272].

Taken together, these data would point to the detrimental effect of fructose compared to glucose in terms of hepatic and lipid metabolism. However, there is controversy between different studies, probably due to differences in the doses of sugars administered and their form of administration (oral, intraperitoneal injection, etc.). Many of the above-mentioned studies were performed using supra-doses of fructose in rodents. Since humans typically do not consume fructose as a single sugar, and it is frequently consumed in the form of HFCS, the direct relationship with the real effect of fructose human consumption is not entirely clear. Therefore, more detailed studies on the pattern of sugar consumption in humans should be carried out.

### 4.5. Impact of Excessive Dietary Sugars Consumption on Incretin Secretion

There are many associations reported between high-sugar consumption and the development of pathologies such as diabetes, obesity, and MetS [10,273,274,275]. These associations are mainly due to the current consumption of sugar-sweetened beverages, whose main sweetener is the HFCS. HFCS represents >40% of caloric sweeteners and its consumption has been increased by >1000% between 1970 and 1990. This sugar overconsumption can lead to important changes in the secretion of gut hormones, and therefore, lead to central effects that affect appetite and satiety control.

Many authors have focused their studies on the effect that different GIs and GLs have on incretin secretion. Runchey et al. observed that 28-days consumption of a high-GL diet in weight-maintained healthy individuals led to statistically significant increased post-prandial GIP and lower GLP-1 concentrations compared with low-GL diets [276]. However, other authors did not corroborate these findings clearly. One study performed in healthy sedentary women reported that GLP-1 concentrations did not differ significantly following high- or low-GI meals [277]. In the same way, another study in overweight subjects observed no differences in GLP-1 concentrations when comparing consumption of low- and high-GI beverages [278]. Other authors suggest that the rate of small intestinal glucose exposure (i.e., GL) is a major determinant of the magnitude of the incretin effect, since they observed that the incretin effect was stronger when they administered larger intraduodenal glucose load [279].

However, it is necessary to be cautious with this upregulation of incretins in response to high-sugar diets, because it has been described that in diabetic patients, who have increased levels of incretins, a reduced incretin effect is observed, which suggest the development of an “incretin resistance” process [280,281,282,283].

## 5. Future Directions

During human evolution, ancestral human diets contained low carbohydrate levels and most of the sugars were derived from fruits and honey. In the last century, changes in lifestyle, nutritional habits in the world population, and the abusive use of sweeteners by the food industry have dramatically increased dietary sugar consumption, particularly constituent monomers, such as glucose and fructose, and fructose-based sweeteners. International and national health organizations have called attention into this issue and recommend reductions in sugars consumption due to concerns in their potential role as risk factors for developing human diseases such as obesity and T2DM.

In the last decades, the scientific community has made great efforts to understand intestinal sugar absorption, identifying molecular and physiological mechanisms of fructose and glucose sensing and transport. In the case of fructose metabolism, the current notion that fructose is mainly metabolized by the liver has been challenged, and the new paradigm proposes that the small intestine shields the liver from toxic fructose exposure. This provocative view of intestinal fructose metabolism is awaiting confirmation in humans. Similarly, the finding that humans can synthesize fructose by the polyol pathway leaves open the question about the significance of this pathway in human metabolic diseases. In addition, more meta-analysis studies should be performed to clearly demonstrate the causal role of dietary fructose and glucose in developing human metabolic diseases.

On the other hand, the role of intestinal glucose metabolism on the etiology of hyperglycemia remains incompletely clarified. Despite studies relating chronic hyperglycemia with impaired glucose transport and metabolism in the small intestine, more studies in humans are required to reveal if chronic hyperglycemia is a cause or consequence of impaired glucose homeostasis in the small intestine. The identification of molecular mechanisms by which glucose and insulin regulate SGLT1 have set this transporter, and its potential role in the physiopathology of hyperglycemia and intestinal insulin resistance, in the spotlight. In this line of thinking, further research is required to demonstrate the efficacy of SGLT1 inhibitors in the treatment of T2DM and obesity. Likewise, it remains to be clarified whether the apical localization of GLUT2 in response to high glucose levels in obese and/or diabetic patients is an adaptive mechanism to protect the body from excessive glucose concentrations, or if it is a consequence of hyperglycemia and insulin resistance.

Finally, the differentiated effects of glucose and fructose on eating behavior and impaired cognitive function observed in rodent models are difficult to extrapolate to humans due to the use of extremely high-fructose diets or unusual glucose to fructose ratio. To clarify the causality of fructose in human eating disorders leading to metabolic diseases, it is necessary to develop new research tools and experimental approaches in humans.
